# Bambara Groundnut: An Underutilized Leguminous Crop for Global Food Security and Nutrition

**DOI:** 10.3389/fnut.2020.601496

**Published:** 2020-12-10

**Authors:** Xin Lin Tan, Susan Azam-Ali, Ee Von Goh, Maysoun Mustafa, Hui Hui Chai, Wai Kuan Ho, Sean Mayes, Tafadzwanashe Mabhaudhi, Sayed Azam-Ali, Festo Massawe

**Affiliations:** ^1^Future Food Beacon Malaysia, University of Nottingham Malaysia, Semenyih, Malaysia; ^2^School of Biosciences, University of Nottingham Malaysia, Semenyih, Malaysia; ^3^Plant and Crop Sciences, School of Biosciences, University of Nottingham, Sutton Bonington Campus, Leicestershire, United Kingdom; ^4^Center for Transformative Agricultural and Food Systems, School of Agricultural, Earth & Environmental Sciences, University of KwaZulu-Natal, Scottsville, South Africa

**Keywords:** Bambara groundnut, legume, food security, nutrition, underutilized crops, dietary diversification, food systems

## Abstract

Rapid population growth, climate change, intensive monoculture farming, and resource depletion are among the challenges that threaten the increasingly vulnerable global agri-food system. Heavy reliance on a few major crops is also linked to a monotonous diet, poor dietary habits, and micronutrient deficiencies, which are often associated with diet-related diseases. Diversification—of both agricultural production systems and diet—is a practical and sustainable approach to address these challenges and to improve global food and nutritional security. This strategy is aligned with the recommendations from the EAT-Lancet report, which highlighted the urgent need for increased consumption of plant-based foods to sustain population and planetary health. Bambara groundnut (*Vigna subterranea* (L.) Verdc.), an underutilized African legume, has the potential to contribute to improved food and nutrition security, while providing solutions for environmental sustainability and equity in food availability and affordability. This paper discusses the potential role of Bambara groundnut in diversifying agri-food systems and contributing to enhanced dietary and planetary sustainability, with emphasis on areas that span the value chain: from genetics, agroecology, nutrition, processing, and utilization, through to its socioeconomic potential. Bambara groundnut is a sustainable, low-cost source of complex carbohydrates, plant-based protein, unsaturated fatty acids, and essential minerals (magnesium, iron, zinc, and potassium), especially for those living in arid and semi-arid regions. As a legume, Bambara groundnut fixes atmospheric nitrogen to improve soil fertility. It is resilient to adverse environmental conditions and can yield on poor soil. Despite its impressive nutritional and agroecological profile, the potential of Bambara groundnut in improving the global food system is undermined by several factors, including resource limitation, knowledge gap, social stigma, and lack of policy incentives. Multiple research efforts to address these hurdles have led to a more promising outlook for Bambara groundnut; however, there is an urgent need to continue research to realize its full potential.

## Introduction

Eliminating hunger requires an adequate intake of energy and nutrients. Providing a healthy diet requires a food-based approach to improving diet and nourishing individuals. Despite the rich agrobiodiversity on Earth, humanity has evolved to rely on a few crops for nourishment. The last few decades have seen a global increase in the supply of dietary energy, through increased yield and production worldwide (1). However, this does not translate to the nutritional quality of the food we consume, nor does it ensure availability, accessibility, and affordability of food to vulnerable populations. The recent decades have seen an increase in prevalence of hunger, childhood overweight, and adult obesity (2). Should we continue with our current production and consumption patterns, we are unlikely to achieve the UN Sustainable Development Goal (SDG) of Zero Hunger by 2030 (2). Factors such as population growth, urbanization, and changes in dietary pattern toward resource-intensive foods are driving the demand for increased food production (3). Pest and disease outbreaks, resource depletion, regional conflicts, and climate change are set to further undermine the capacity of the food system and exacerbate the situation (2, 3). To meet the SDG of zero hunger by 2030 and to end malnutrition in all its forms, the target is to increase the availability and accessibility to nutrients, not just calories. Adoption of a diversified healthy diet, with emphasis on affordable nutrient-rich plant-based foods such as fruits, vegetables, whole grains, and legumes can contribute to sustainable food and nutrition security (2, 4) and to the achievement of SDG2.

Bambara groundnut (*Vigna subterranea* (L.) Verdc) is a legume indigenous to Africa and is cultivated across the semi-arid sub-Saharan Africa region (5). It is a hardy crop and has been recognized as an important nutritious food source when food is scarce (6). This could be attributed to its climate-smart features, including its ability to fix nitrogen, and to grow under adverse environmental conditions such as poor soils and drought (7, 8). This nutrient-dense legume is sometimes termed a “complete food” due to its balanced macronutrient composition. Bambara groundnut contains ~64.4% carbohydrate, 23.6% protein, 6.5% fat, and 5.5% fiber and is rich in minerals (9). It is relatively underutilized compared with major cash crops and has often been associated with small-scale, subsistence farming, with women being the major producers and processors (6, 10). The utilization constraints of Bambara groundnut include the knowledge gap in improved seed system, agronomic practices, processing, and utilization. Genetics, agronomy, and nutritional aspects of Bambara groundnut and its food uses have recently been reviewed by other authors (7, 9, 11, 12). This paper gives an overview of the value chain and discusses the potential role of Bambara groundnut in closing the gaps in the food system to ensure sustainability of food and nutritional security.

## Closing the Food Supply Gap Through Improved Production of Bambara Groundnut

Bambara groundnut is thought to have its center of origin somewhere between West and Central Africa (13). It is grown widely in sub-Saharan Africa and is also present at low levels in Thailand, Malaysia, and Indonesia (7). Higher preference for Bambara groundnut has been observed in dry regions prone to drought (14). This is possibly linked to its ability to produce reasonable yields under such conditions, hence acting as a safety net for farmers. Bambara groundnut production in Africa is reported to be ~0.3 million tons annually with an average of 0.85 t/ha, although the yield potential is reported to be over 3 t/ha (5, 15). Nigeria is regarded as the largest producer of Bambara groundnut with a mean production of 0.1 million tons, followed by Burkina Faso 44,712 tons, and Niger 30,000 tons (5).

### Genetic Diversity and Implications—Traditional Landraces vs. Modern Varieties

Most germplasm planted by famers is in the form of landraces with high genetic variability. This is reflected by the wide variations in morphological (16) and nutritional (9) traits across Bambara groundnut landraces. Genetic variability can act as a form of insurance for farmers as some members of the landrace population can provide local adaptation, stress tolerance, and yield stability (17), thus giving farmers a higher chance of obtaining some form of seed yield in times of drought or other stresses.

High genetic variability observed in landraces also lends itself to high potential for crop improvement in Bambara groundnut. Most of the currently grown improved varieties of Bambara groundnut are generally landraces selected for improved yield, seed and flour quality, and drought tolerance (18). Bambara groundnut is usually sown as a minor crop, intercropped with other staples, by small holder African farmers for household consumption (5). For this reason, coupled with low current market demand for the crop, yield stability is seen to be a more important aspect for landrace improvement than grain yield in order to ensure food security. Besides, the genetic diversity preserved in the gene pool allows some of these accessions to be developed into high protein and high oil cultivars (9), suggesting its potential in contributing to nutritional security in the region. Breeding improvement efforts of the promising landraces could consequently lead to improved profitability of the crop as well as adoption of the crop in diverse Bambara groundnut growing regions.

### Productivity Traits and Agroecological Adaptation

Variation in Bambara groundnut productivity has been attributed to agroecological factors, such as climate (19), soil fertility (20), water availability (21), and daylength (22). Nonetheless, it has been shown to exhibit adaptability across different regions under diverse growing conditions. For instance, the crop exhibits tolerance to soil acidity and low soil fertility (23), as well as adaptability to the tropical degraded acidic soils (24). Despite being classified as a facultative short-day crop for pod set (22), many landraces have adapted to regions with a broad range of daylengths. Physiological experiments have also revealed good recovery qualities when the crop is subjected to water stress (25). Its yield is reported to be well above those of chickpea and similar to groundnut cultivars under comparable drought stress conditions (26, 27). This indicates that selection for drought tolerance is key considering that the crop is generally cultivated in arid to semi-arid regions of sub-Saharan Africa. Limited studies indicated that, although field drought conditions reduce the seed yield in Bambara groundnut, there is no effect on the nutritional quality of the seed (28). This trend has been observed in limited landraces and also in common bean (*Phaseolus vulgaris* L.) (29), but further studies on Bambara groundnut would be required to confirm this hypothesis.

Some areas where Bambara groundnut is grown have poor soils that are lacking in nitrogen. Most farmers in those regions do not apply synthetic fertilizers to their crops because the costs are often prohibitive (30). Bambara groundnut, as other nodulating legumes, can fix atmospheric nitrogen to replenish soil nitrogen, hence making it a potential companion crop for intercropping and rotational systems. It is often intercropped with cereals and root crops that can provide a significant amount of the calorie intake (31). Its incorporation into crop rotation cycles can help to maintain soil fertility and break the cycles of pests and diseases, which is advantageous to resource poor farmers who might generally be unable to afford fertilizers and pesticides (32). Incorporation of Bambara groundnut in intercropping system with maize has been shown to increase the productivity of maize (33), indicating its potential contribution toward agrobiodiversity and subsequently food security. Varying rates and amount of nitrogen fixation have been observed for different Bambara groundnut accessions (24), and the enhancement of symbiotic nitrogen fixation was indicated to potentially increase its yield (34). The variability of nitrogen fixing capacity among Bambara groundnut landraces offers room for cultivar improvement and a positive correlation with yield would be an ideal scenario for the breeder.

## Closing the Nutrient Gap Through Enhanced Utilization of Bambara Groundnut

### Bambara Groundnut as a “Complete Food”

There is a growing trend toward increased consumption of plant-based diets, resulting in a need for more plant-based protein foods. Bambara groundnut is the obvious crop to consider. It serves as an important source of essential nutrients in areas where animal protein is scarce (35). The nutritional composition of Bambara groundnut has earned it the reputation of being a complete food, and this will be explored further in subsequent sections.

#### Balanced Macronutrient Composition

##### Carbohydrates

Carbohydrates are the most abundant macronutrient in Bambara groundnut, accounting for up to 64.4% of the total dry weight of the seed (9). The majority of the carbohydrate fraction is complex oligosaccharides and polysaccharides, of which starch accounts for up to 49.5% of the total carbohydrates. The reported starch content of Bambara groundnut seeds varies considerably (22 to 49.5% of dry seed weight), depending on genetic and environmental factors, stage of maturation, and method of analysis (12). Amylose represents 19.6–35.1% of the total starch content, while the rest of the constituents consist primarily of amylopectin and a small quantity (1–2%) of protein, lipid, and ash (12). Raw Bambara groundnut has a higher proportion of slowly digestible starch (SDS) and resistant starch (RS) than rapidly digestible starch (RDS) (36), implicating poor digestibility. Nonetheless, cooking can substantially increase the RDS fraction (37), thereby improving digestibility and carbohydrate availability.

##### Protein

The protein content of Bambara groundnut ranges from 9.6 to 40% (11), with an average value of 23.6% (9). This variation is also attributed to differences in genetic background, growing conditions, and analytical techniques used for estimation (e.g., nitrogen conversion factor) (10, 35). Storage proteins are the predominant protein fractions in Bambara groundnut, of which vicilin (7S) is reported to be the major constituent, followed by legumin (11S) (38).

High protein content is a desirable trait in foods, but the importance of protein quality, which is determined by both amino acid composition and protein digestibility, should not be overlooked. Variability in amino acid profile between cultivars of Bambara groundnut is evident. In general, most studies report glutamic acid to be the most abundant amino acid in Bambara groundnut, suggesting its potential to be isolated for use as a flavoring agent. Out of the essential amino acids, leucine and lysine are present at a higher concentration while methionine is the lowest (39–41). Phenylalanine, valine, histidine, and isoleucine were also reported to be present in high concentrations, while tryptophan has been found to be the limiting amino acid (37, 39). Its lysine-rich, methionine-poor composition makes Bambara groundnut a good complementary protein source to cereals, which are often deficient in lysine but rich in methionine (35). The *in vitro* protein digestibility (IVPD) of raw and cooked Bambara groundnut varies between 70 and 81% and 82 and 87.5%, respectively (37, 42). The increase of IVPD after cooking is attributed to the destruction of heat labile antinutritional factors (ANFs) and fragmentation of native proteins into smaller polypeptides, subsequently improving enzyme accessibility and protein bioavailability.

##### Lipids

There is considerable variation (1.4 and 9.7%) in the reported values of lipid content in Bambara groundnut (38, 39). The majority of fatty acids in Bambara groundnut are unsaturated, predominated by oleic and linoleic acids (omega-6) (39, 43). Palmitic acid is the third most abundant fatty acid, and linolenic acid (omega-3) is present at a low concentration. While having high unsaturated fatty acid content is appealing from a consumer health perspective, it increases the susceptibility of fats to oxidation and rancidity. Therefore, the end uses should be taken into consideration when selecting the desirable trait of lipid composition.

#### Rich in Essential Micronutrients

##### Minerals

The most abundant minerals in Bambara groundnut are potassium, magnesium, phosphorus, zinc, and iron (37, 41, 44). Halimi et al. (9) reported that the levels of these minerals were higher than those found in commonly consumed legumes such as chickpea and mung bean, but they vary by cultivar and growing conditions. The presence of ANFs in the seeds can adversely affect the bioavailability of the minerals. Gwala et al. (45) reported that the concentration and bioaccessibility of calcium, magnesium, iron, and zinc in Bambara groundnut seeds were influenced by factors such as storage period, processing method, location of mineral in the seeds (testa or cotyledons), and the degree and strength of mineral chelation. Despite being a relatively good source of these minerals, it is unlikely that the dietary needs of individuals can be met through consumption of Bambara groundnut alone.

##### Phytochemicals

Bambara groundnut seeds contain phytochemicals such as flavonoids and tannins. These compounds are usually found in the seed coats and are more abundant in seeds with dark or red-colored seed coats. A positive correlation between darkness of seed coat and total phenolic compounds has been established (46). Mubaiwa et al. (47) reported an abundance of the flavonoids epicatechin and catechin in raw and cooked red seed, respectively. Catechin and epicatechin can polymerize to form proanthocyanidins, also known as condensed tannins, which have been associated with nutraceutical properties, such as antioxidant, cardioprotective, antitumor, and neuroprotective properties (48). Antioxidant properties have been reported in brown and red Bambara groundnut seeds, levels of which were comparable with commonly consumed legumes, but inferior to the powerful antioxidant ascorbic acid (49, 50). Despite the positive health outcomes associated with consumption of phytochemical compounds, their antinutritional implications should not be overlooked.

#### Other Important Functional Properties

##### Dietary Fiber

Bambara groundnut contains appreciable levels of dietary fiber in the form of RS and non-starch polysaccharides. The concentration and composition of dietary fiber are influenced by maturity stage and processing methods (39). Total dietary fiber content of Bambara groundnut ranges from 1.4 to 10.3%, of which insoluble fiber represents a higher fraction than soluble fiber (9). The relatively high proportions of SDS, RS, and dietary fiber in Bambara groundnut reduce the rate of digestion and lower the postprandial glycemic response, rendering Bambara groundnut a low glycemic index (GI) food (36). From one point of view, it is advantageous to encourage the consumption of low GI foods as these confer numerous health benefits, e.g., lowering postprandial blood glucose and insulin levels, regulating appetite, and reducing the risks of obesity and other non-communicable diseases. Conversely, the increased consumption of flatus-causing non-starch polysaccharides has been associated with irritable bowel (51). More importantly, from a nutritional security point of view, non-digestible dietary fibers can bind to minerals and and form a physical barrier to digestive enzymes, thus reducing the bioavailability of essential minerals (52).

### Processing of Bambara Groundnut to Increase Nutritive Value and Utilization

#### Antinutritional Factors

In common with other legumes, several ANFs have been identified in Bambara groundnut. Their presence can negatively affect the digestion and bioavailability of essential nutrients. The commonly reported ANFs in Bambara groundnut include condensed tannins, phytic acid, and trypsin inhibitor. Condensed tannins are mainly located in the testa and are more abundant in the darker-colored seeds (53). Despite having an antioxidant capacity, these polyphenolic compounds can form indigestible complexes with dietary minerals, starch, and proteins, thereby reducing their bioavailability (51, 54). Binding with proteins can inhibit the activity of digestive enzymes. Tannin compounds can also impart bitterness and astringency to the food (48), thereby affecting palatability. Phytic acid is more abundant in the seed cotyledon, where it serves as a phosphorus reserve for the plant (52). At physiological pH, the highly charged phosphate groups have a high tendency to chelate to mineral cations and form stable, indigestible complexes (55). Phytic acid can also crosslink with dietary proteins, starch, and digestive enzymes, thus impairing the bioavailability of nutrients (51, 52). However, it is worth noting that phytic acid has been reported to exhibit antioxidant and anticancer properties, suggesting its potential health-promoting properties (51). The major enzyme inhibitor reported for Bambara groundnut is trypsin inhibitor (55). Inhibition of protease can negatively affect protein digestion and subsequently impede its absorption. Furthermore, low trypsin level can result in increased pancreatic secretory activity, thereby causing pancreatic hypertrophy (43). The reported levels of ANFs among different Bambara groundnut cultivars vary widely (condensed tannins, 0.0011–18.61 mg/g; phytic acid, 1.10–15.11 mg/g; trypsin inhibitor, 0.06–73.40 TI mg/g). These differences are attributed to genetic and environmental factors, as well as extraction and analytical methods (54, 55).

Some forms of dietary fibers are also considered to have antinutritional properties. Pectins can bind to metal cations such as calcium, zinc, and iron, which, not only reduces mineral bioavailability, but affects the cookability of the legume (52). Raffinose and stachyose, the flatus-causing alpha-oligosaccharides, are also present in Bambara groundnut (43, 55). Other ANFs such as oxalate, hydrogen cyanide, and saponins have also been detected in Bambara groundnut (43, 46, 56).

Certain food processing methods are effective at lowering the ANFs, and this will be discussed in the following section. It is possible that the inherent levels of ANFs present in raw beans could be reduced by plant breeding (57), which would be advantageous for improved utilization of the legumes and in their contribution to enhanced nutritional security. However, gains made in improving the nutritional value through reduction of the antinutritional compounds, may be lost through increased susceptibility to pests and diseases during production and subsequent storage of the seeds. This is because these components are plant secondary metabolites that provide some resistance to stress, pests, and pathogens, therefore, reducing the levels may result in a compromised defense system (57).

#### Traditional Processing Methods

If not eaten fresh, Bambara groundnut is dried postharvest for long-term storage. Drying is an effective food preservation technique to prolong the storage period and ensure food availability during food shortages (58). Prior to consumption, the dry seeds are either rehydrated by soaking in water or milled in the dry form into flour. Most of the pretreatments or processing have an impact on the nutritional, sensory, and functional properties of the seeds. Traditional processing of Bambara groundnut involves basic equipment and can be carried out at the household level. Some of these processes have the potential to be mechanized and industrialized to improve the cost effectiveness, process efficiency, and product uniformity, while creating employment opportunities and providing income for rural people (58). The following section describes some of the traditional, often essential, processing stages of Bambara groundnut, and the impact on nutritional, processing, and eating quality.

##### Dehulling

The seed coat, or testa, is sometimes separated from the cotyledons before further processing. Since a high proportion of the antinutritional components are present in the testa, dehulling can improve the digestibility and nutritional value of the seeds, in particular through increased mineral and protein availability (40, 59). Removal of the testa also reduces the dietary fiber content (45, 52), which can have both negative and positive implications, depending upon the nutritional status of the consumer. With respect to sensory attributes, removal of the highly pigmented seed coats, which are rich in tannins and fibers, has been shown to improve the appearance, texture, and taste of Bambara groundnut products (53). Other implications of dehulling include increased leaching of minerals during soaking and cooking, which negatively impacts on the nutritional quality, and shortened fermentation time which is advantageous from a utilization point of view (40).

##### Milling

Dried Bambara groundnut can be ground into flour to improve its versatility (11). However, at the small scale, milling is laborious and time consuming due to a phenomenon described as “hard-to-mill” (10). The disruption of cell wall structure, through milling or other abrasive processing activity, can increase the availability and digestibility of nutrients, starch, and protein in particular (60). Increased interaction between starch, protein, and cell wall materials also results in structural and functionality changes (61). From a negative point of view, milling could increase interactions between minerals and ANFs, thus reducing their bioavailability (62). In terms of food security, the hard-to-mill attribute is advantageous in that it allows dried seeds to be stored for very long periods as they are impervious to water and resistant to pest and insect attack. However, from the utilization aspect, these inherent difficulties incur increased energy costs for processing and may deter potential end users from choosing Bambara groundnut as a raw material, despite its nutritional and agronomic advantages.

##### Soaking

After drying, Bambara groundnut seeds are typically rehydrated by soaking in water for 12–24 h before cooking. Soaking also has positive and negative impacts on the nutritional value of the seeds, primarily through leaching of nutrients and ANFs into the soaking water (42, 52). The rate and degree of leaching are influenced by the binding strength of the biomolecules to the intracellular matrix (45), which can be manipulated by the temperature and pH of the soaking liquid. Studies reported higher loss of trypsin inhibitor and tannins in Bambara groundnut during hot water soaking, but the reverse was observed for phytate and oxalate (63, 64). Soaking also facilitates the subsequent processing of Bambara groundnut. Increasing soaking temperature (up to 60°C) improved water absorption rate and dehulling efficiency and reduced dehulling loss (61). Regarding functional properties, presoaking Bambara seeds before milling results in flour with higher foam capacity and improved pasting properties (10, 64). From a food and nutritional security perspective, soaking is a low-cost, low-energy processing stage that can significantly improve the utilization of Bambara groundnut.

##### Germination/Malting

The nutritional value of Bambara groundnut seeds can be manipulated by germination. The process of soaking followed by sprouting for up to 72 h (65) reduces the carbohydrate and lipid content of the sprouts (66), while at the same time enhancing the protein content, amino acid profile, and IVPD (43, 67). Reduction of ANFs, such as tannins, trypsin inhibitor, oxalate, oligosaccharides, and saponin, is due to leaching during soaking (43, 66). Sprouting is also beneficial to the dehulling process (63) as the seed coat splits open during sprouting.

##### Fermentation

Fermentation is another traditional, low-technology processing option that can be used to enhance the nutritional value of Bambara groundnut. Typically, the process involves soaking of the whole seeds, followed by dehulling, cooking, and wrapping in banana leaves before fermenting for about 4 days (40). Starter cultures may sometimes be added (44) to enhance the fermentation. The positive impacts of fermentation are a breakdown of the flatus-forming non-digestible oligosaccharides and polysaccharides into digestible simpler carbohydrates and a reduction in ANFs and phenolic content (40). The production of yogurt from extracted Bambara groundnut milk is reported to improve protein content and digestibility while lowering the phytate content (68).

##### Boiling

Bambara seeds are often cooked in excessive water for variable periods of time until the desired texture is attained. In common with the other treatments, genetic variability, physicochemical properties, age of the seed, and storage conditions can affect the time taken to reach the desired end point. In the presence of water, thermal treatment leads to starch swelling and gelatinization, protein denaturation, solubilization of water-soluble pectins, and eventually cell separation (60). The effects of boiling on nutritional quality of Bambara groundnut vary with cultivar, pretreatment applied, and the length of cooking time. However, most studies report that boiling has a positive impact on nutritional quality through the destruction of ANFs (53, 56, 64) and improved *in vitro* digestibility of starch and protein (37, 42). It is likely that improved digestibility of protein is due to destruction of ANFs, which are more susceptible to wet-heat than dry-heat, thereby releasing protein bound to them; whereas improved starch digestibility is due to the disruption of starch granules, consequently increasing amylolytic attack, and hydrolysis.

The various processing stages applied to Bambara groundnut, at small and larger scale, have positive and negative implications for its food and nutritional security. The presence of various ANFs and the indigestible nature of the raw seeds mean that processing is an essential stage for all legumes. From a food and nutritional security point of view, it is important to select a combination of processes that enhance the digestibility of the macronutrients, while at the same time minimizing losses of both macronutrients and minerals. When selecting or recommending particular processing methods, it is essential to consider the energy costs of each one, as these can be prohibitive to the utilization of the crop by resource-poor end users.

#### Traditional Food Uses

**Figure 1:**
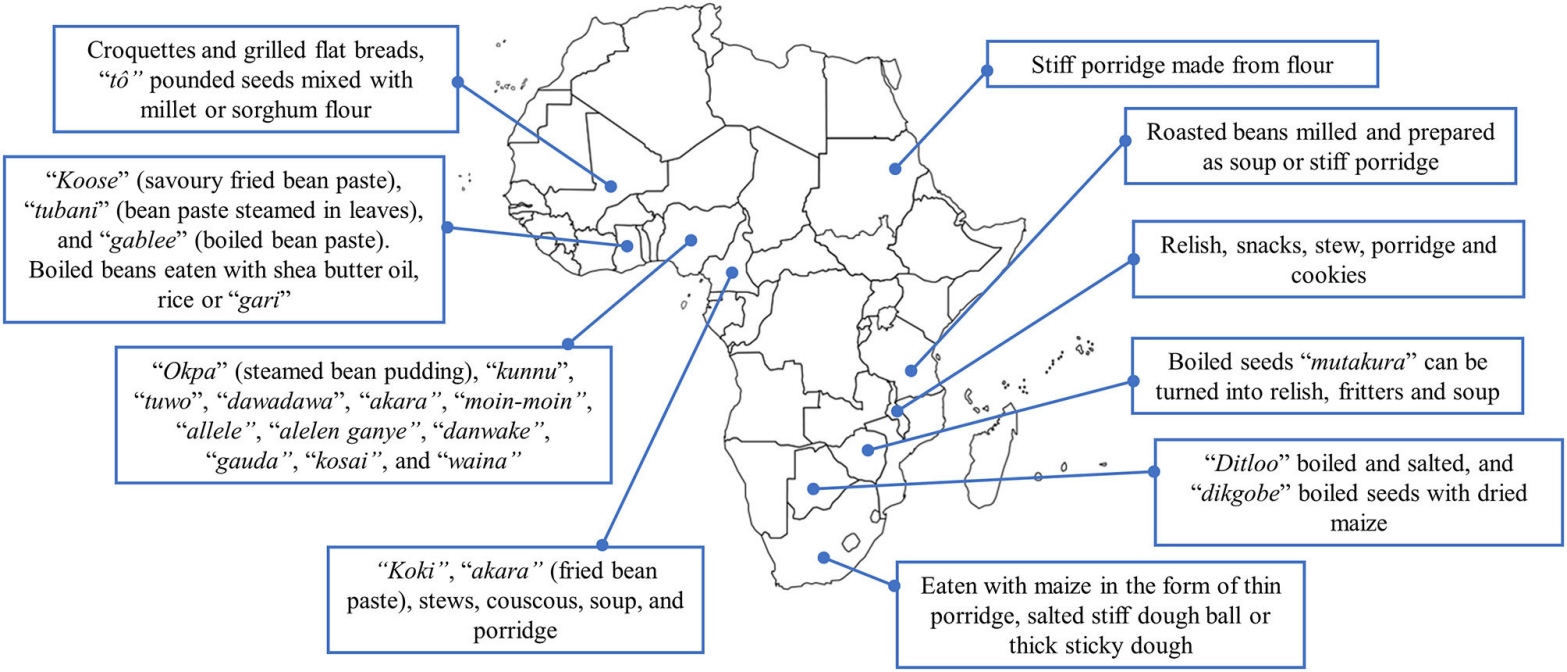
Traditional local delicacies in different African countries prepared from Bambara groundnut.

Bambara groundnut is commonly consumed as snack food after roasting or boiling (14, 69). The seeds and the flour have also been used to produce a myriad of traditional foods in different parts of Africa (Figure 1) (6, 14, 53, 56, 70). During the preparation of local delicacies, Bambara groundnut is often paired with cereals such as maize and millet (6, 69), which is beneficial in improving protein quality. In Nigeria, the popular “*okpa”* (steamed Bambara groundnut pudding), which is made from Bambara groundnut flour and red palm oil, plays an important role in contributing to dietary protein and vitamin A intake among school children (71).

### Advanced Processing Technologies and Potential Food Uses of Bambara Groundnut

#### Advanced Processing Technologies

In addition to the traditional low-cost processing technologies, which are essentially used to make the seeds edible and fit for consumption, more advanced forms of processing can be employed to improve nutritional quality, modify the physicochemical characteristics, and expand the range of value-added products for increased utilization. Irradiation, infrared heating, and autoclaving are among these more advanced techniques. Unlike traditional processing techniques, which can be employed at household level, these technologies require more sophisticated equipment and therefore are carried out on a larger scale.

Despite being a non-thermal processing method, gamma-irradiation causes starch degradation or pregelatinization in Bambara groundnut, thus reducing the cooking time of the seeds (72). Shorter cooking time could also be due to increased cell wall permeability to water and/or heat as a result of damaged microstructure (73). Irradiation also affects protein structure and conformation, thus altering the functionality of the flour (72).

Infrared heating (micronization) can also result in reduction in cooking time of Bambara groundnut (74), which not only helps to save energy and water but also has knock-on positive impacts on the retention of nutrients. This instant heating process, which causes starch gelatinization and protein denaturation, could enhance the utilization of Bambara groundnut seeds and flour for production of a diverse range of convenience products such as partially cooked seeds or instant flour (75).

The use of high-pressure heating, via autoclaving, is useful for improving the nutritional value of the cooked seeds. The application of heat under high pressure is effective at deactivating ANFs, thus increasing the digestibility of proteins and starch (43). The high-temperature process can reduce the functionality of proteins, as measured by a reduction in foaming and emulsifying properties (64), which will limit the use of Bambara groundnut in food applications where emulsification and/or the ability to form a stable foam are essential attributes. From a nutritional security point of view however, any process that can improve the nutritional quality through improved digestibility and removal of ANFs, at the same time as reducing the cooking time and energy requirements, is a beneficial process.

#### Bambara Groundnut in Processed Foods

Advanced processing technologies and enhanced insights into food science, coupled with increased consumer demand for convenience food have led to increased availability and accessibility to processed foods at lower prices (76). While processed foods offer numerous advantages to consumers such as convenience, diverse food choices, and stability of food supply (76), the ultra-processed foods are often stripped of nutrients and are energy dense. Excessive consumption of empty calories can have negative impacts on health. To replace the micronutrients that are removed by processing, public health initiatives make the fortification of some food commodities with vitamins and minerals mandatory in most countries. Increasingly, alternative ingredients are being used to fortify staple foods, rather than relying on premix vitamins and minerals. This approach confers additional benefits to the consumer and producer—it offers a more holistic approach to food fortification or enrichment, utilizing otherwise underutilized plant species, and diversifying the diet with more than just the specific mineral or vitamin. Bambara groundnut has the potential to serve as an ingredient for food fortification due to its affordability, versatility, nutritional quality, and sensory acceptability.

Nwadi et al. (11) recently reviewed studies on the application of Bambara groundnut flour in a number of products including snacks and pastries, breakfast cereal and pasta, traditional foods, composite flour, complementary food, and milk and yogurt. To summarize, most studies (77–79) reported that the protein content of the Bambara groundnut flour-incorporated products increased with the supplementation level, but sensory attributes were negatively affected. This paper discusses a few points that were not covered by Nwadi et al. (11) review.

##### Staple Foods: Breads and Wheat noodles

Bread is one of the staple foods consumed on a global scale. It is commonly made from wheat flour, which is deficient in lysine. Complementing with legume flour or extracted legume protein can therefore improve its nutritional quality. However, consumers have come to expect leavened bread to have an airy texture, which is the result of gas being trapped inside a gluten network. Like other legumes, Bambara groundnut flour lacks gluten. Inclusion of Bambara groundnut flour into bread dough therefore has an adverse impact on the texture of the bread and this will limit the amount that can be used to substitute wheat flour. Reports on the use of Bambara groundnut flour in bread highlight impacts on protein weakening, increased dough development time, decreased dough consistency, stability and extensibility, water absorption, and loaf volume (80, 81). These observations were attributed to an impaired development of the gluten network due to dilution of gluten. Nonetheless, the dough rheology and physical characteristics of Bambara groundnut flour-incorporated dough can be improved by addition of active surfactants such as pectin and emulsifiers (78), thereby improving the organoleptic properties of breads.

Wheat noodles are staple in many Asian countries and are gaining popularity in other parts of the world. Gluten is also important in the production of noodles but not as critical as for a leavened bread. Wheat-based noodles are increasingly being fortified with ingredients such as spinach, pumpkin, and sweet potato to improve the nutritional value. Bambara groundnut flour has been included in wheat-based noodles with varying degrees of success (41, 82).

In general, addition of Bambara groundnut flour into bread and noodles improved the protein quality, reduced ANFs, and increased mineral contents (41, 77, 81). The level of substitution of wheat flour could be the key to consumer acceptability. An appropriate substitution level can help to ensure improved nutrition without compromising consumer acceptability (41, 81), thus making these products a promising food vehicle to combat nutrient deficiency. An alternative, more promising option for developing this area is to change consumer expectations, by marketing Bambara groundnut-enriched loaf/noodle as a novel and nutritious alternative product (41).

##### Snacks: Crackers/Biscuits/Extruded Products

Popular snack foods, such as crackers, biscuits, and extruded products, are typically made from wheat, rice, or maize. They are potential foods for fortification programs targeting school children and adult alike. Studies on the inclusion of Bambara groundnut flour in these products showed variable results in terms of their physical and functional properties (83–85). These variations could be due to different formulations and production methods used, which might also explain the differences in nutritional and sensory qualities among these products. In order to maximize the potential of Bambara groundnut-enriched snacks in improving nutritional security of the population, local consumer preferences should be taken into consideration during product development.

##### Complementary/Weaning Foods

Traditional weaning foods in Africa are often prepared from low-cost but highly accessible ingredients such as cereals, roots, tubers, and legumes (86, 87). They are often nutrient poor, characterized by high levels of starch, fiber, and antinutrients, but with inadequate levels of essential amino acids and micronutrients. Inappropriate processing methods also lead to poor texture and nutrient bioavailability (86). Several studies have successfully developed complementary foods containing Bambara groundnut and reported enhanced nutritional quality: Bambara groundnut-enriched maize “*ogi*” showed increased protein, ash and fat contents and high consumer acceptability (88); banana and fermented Bambara groundnut flour mix at 60:40 ratio showed comparable nutritional quality with commercial infant formula (89); and maize-Bambara groundnut complementary food fortified with micronutrients showed acceptable micronutrient levels to meet infant daily requirements (90). To conclude, its low-cost, nutrient-dense features allow Bambara groundnut to be a viable alternative ingredient in enriching infant food products.

##### Beverages: Milk and Fermented Drink

Inaccessibility of dairy milk in some countries, specific health-related dietary requirements and the trend toward plant-based diets are factors that are driving the surge in demand for vegetable milk (91, 92). Bambara groundnut has the potential for the production of vegetable milk and yogurt [reviewed by Murevanhema and Jideani (69)]. The development of shelf-stable spray-dried milk powder with acceptable hydration properties has been reported (93). Several reports refer to the nutritional and/or sensory properties of Bambara groundnut milk and yogurt (68, 92, 94). Overall, it can be concluded that despite its high nutrient content, there remains works to be done to optimize the sensory and physicochemical properties of Bambara groundnut milk to gain wider consumer acceptance.

*Amahewu*, or *mahewu*, is a popular fermented drink traditionally made by fermenting sorghum or maize flour. It is non-alcoholic and is sometimes used as a weaning food (44). However, it is nutrient poor and is often characterized as lacking essential amino acids (67). Substituting the conventional ingredients with Bambara groundnut reduced phytate content while improving its protein quality and sensory acceptability (44, 67).

#### Bambara Groundnut as a Functional Ingredient

In addition to providing essential nutrients, both the starch and protein of Bambara groundnut have functional properties, which may find wide application for food and non-food uses. Understanding and improving the functional properties of Bambara groundnut starch and protein isolate may increase the potential application and end-use of the crop, which *may* translate into an increased demand for the crop and benefits to the producer. However, it is prudent to point out that the relationship between increased utilization and producer benefits is far from simple—it is complex, fraught with issues of sovereignty and equity, and with no guarantee of improved livelihoods for the producer.

##### Native Starch

Starch is one of the most widely used and adaptable polysaccharides; in addition to providing energy, within the food industry, starch is used variously as a thickener, gelling agent, stabilizer, and humectant; and for non-food uses such as a replacement for polystyrene and plastic in disposable packaging material, plates, and cutlery, to name but a few. To fully maximize the potential application of Bambara groundnut starch, it is essential to understand the structure and physicochemical properties of the native starch.

The composition, physicochemical properties, and modification of starch has been reviewed by Oyeyinka and Oyeyinka (12). Bambara groundnut starch granule is characterized by spherical, polygonal, irregular, or oval-shaped granules with smooth surface and a diameter from 6 to 35 μm (12, 95). Both the major starch constituents, the amylose and amylopectin fractions, influence its physicochemical and functional properties, which in turn affect its application.

Bambara groundnut starch has poor swelling capability compared with conventional starches such as potato, corn, and cereal starches (12). This could be attributed to its relatively high amylose content, which results in a more rigid granular structure and therefore restricted swelling. The swelling power of Bambara groundnut starch increases with temperature, peaking at 80–90°C, and decreasing thereafter (96). This temperature range corresponds to its gelatinization temperature range, beyond which the starch granules rupture and the contents leach out, leading to inhibition of water uptake and swelling ability (97). The peak gelatinization temperature and enthalpy of gelatinization of Bambara groundnut starch are higher than most of those reported for cereal and tuber starches (98), indicating its thermal stability. Bambara groundnut starch also exhibits a relatively high pasting temperature that is comparable with other legumes (97), while its pasting viscosities show huge variations (12). This could be due to differences in cultivar, experimental condition, for instance starch concentration and purity, and the analytical equipment used, thus making it difficult to compare the results (99).

Its poor functionality, e.g., low swelling capacity and poor pasting properties gives Bambara groundnut native starch limited applications as a functional ingredient. Possible application may include products in which restricted swelling and high thermal stability are desirable.

##### Modified Starch

Native Bambara groundnut starch can be modified to improve and diversify its behavioral characteristics. Modifications can be made physically or chemically. Physical modification, which is considered a safer modification approach, is associated with alteration of the starch granules by heat application (12, 98). Heat treatment leads to intragranular molecular reorganization of starch, thus leading to variable effects such as increased gelatinization and pasting temperatures, and reduced swelling power, solubility, and pasting viscosity (12). On the other hand, chemical modifications alter Bambara groundnut starch structure through introduction or formation of new functional groups (98), thereby affecting its physicochemical properties. Different modification methods (oxidation, acetylation, and carboxymethylation) can have variable effects on the solubility, swelling capacity, pasting properties, and water and oil absorption capacities of Bambara groundnut starch (100, 101). Bambara groundnut starch can also be modified by forming complexes with other components, such as lipids and cyclodextrin. This leads to increased thermal stability, and the resultant starch paste displays higher ability to withstand shear stress with a lower tendency to retrograde (96, 102).

These studies indicate that, with proper selection of modification techniques, Bambara groundnut starch has a great potential to be used as a functional ingredient in food applications such as improving viscosity, mouthfeel, adhesion, and freeze-thaw stability (98). Reduced digestibility of lipid-modified starch (102), which could be due to formation of type-4-resistant starch (103), could provide important nutritional functionality for diabetic patients or in weight management program.

##### Bambara Groundnut Protein Isolate

Bambara groundnut protein can be extracted and used as a functional ingredient in a number of foods. The reported protein content in Bambara groundnut protein isolate (BGPI) ranged from 81.4 to 92.8% (95, 104). Its solubility is pH dependent and has been shown to be higher than mung bean and black bean protein isolates (104). Besides, BGPI displays high thermal stability, which is comparable with mung bean, black bean, and soy protein isolates (38, 104). Studies reported variable results on the physicochemical properties (water and oil absorption capacities, gelation capacity, and foaming and emulsifying properties) of BGPI (105–107). The differences in functional properties of BGPI can be explained by several factors, including the amino acid composition (104), extraction method (38), extraction condition (108), and drying condition (109).

Since the physicochemical properties of BGPI can be altered and improved by various methods, BGPI has the potential to be a useful functional ingredient, especially for those avoiding animal-based products. BGPI exhibits high trypsin inhibitor activity (104), which is undesirable from a nutritional quality point of view, but can be exploited as a preservative. BGPI can act as protease inhibitor to lower proteolysis and improve the gel properties of surimi when applied at an appropriate level (110). The hydrolysates of BGPI have been shown to exhibit potent antioxidant activities, which may find application in food preservation or as a functional food (111). The bioactive peptides were also found to inhibit renin and angiotensin-converting enzyme (ACE), two components known to be associated with hypertension (112).

### Enhanced Production and Consumption of Bambara Groundnut as Food: Challenges and Recommendations

**Figure 2:**
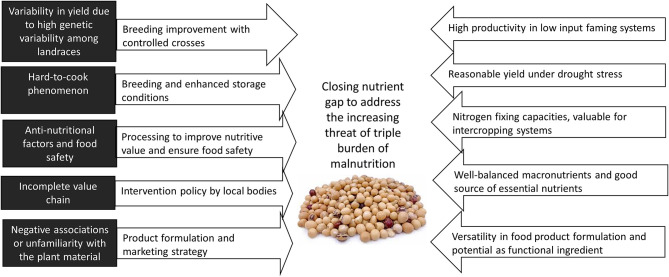
An overview of the challenges and opportunities of utilizing Bambara groundnut in addressing food security and nutrition.

The utilization of Bambara groundnut is challenged by several factors, as summarized in Figure 2. This section focuses on three of the constraints, namely the cookability, bottlenecks in the value chain, and safety issues, and discusses the possible solutions to these challenges.

#### Hard-to-Cook Phenomenon

The hard-to-cook (HTC) trait in Bambara groundnut is one of the major hurdles to its utilization [reviewed by Mubaiwa et al. (113)]. The HTC feature is associated with legume cotyledon resistance to softening during cooking, resulting in a prolonged cooking time to attain a desirable texture. Development of the phenomenon can be hereditary (114), and it can also be induced by extended storage under elevated temperature (>25°C) and humidity (>65%), that is, the ambient storage conditions in humid tropic areas (115). The pectin-cation-phytate model is the most widely accepted postulated mechanism for the development of HTC (114, 116): Pectin is present in the middle lamella where it acts as intercellular cement. Phytic acid, being a powerful chelator for cations, is usually bound to divalent cations especially Ca^2+^ and Mg^2+^. During storage, phytase hydrolyzes the phytate-cation complex, hence liberating the divalent cations; meanwhile, pectin methylesterase removes the methyl esters of pectin, thus producing free carboxyl groups (-COO^−^), which favor binding to the free divalent cations. The formation of insoluble Mg- or Ca-pectate complexes strengthens cell wall structure and cell-cell adhesion, thereby restricting cell separation and bean softening during cooking (117). Mubaiwa et al. (47) also suggested the involvement of phenolic compounds (tannins and hydroxycinnamic acid) in binding to pectin to form insoluble pectate, thereby reinforcing cell adhesion. Another popular hypothesis is cell lignification, which postulates that protein degradation may occur during storage, and the crosslinking between the freed aromatic amino acids and free phenolic compounds in the cell leads to lignin synthesis and causes cell wall toughening (114, 117). Besides, other mechanisms, e.g., lipid peroxidation (118), protein solubilization (119), cell membrane damage, and ion redistribution (120, 121) might also occur during storage, soaking, and cooking. The synergism of these reactions may amplify the effects, eventually leading to HTC phenomenon (116).

HTC phenomenon is reported to have negative impacts on the nutritional quality of Bambara groundnut. Gwala et al. (45) found that aging decreased *in vitro* bioaccessibility of calcium and magnesium in Bambara groundnut seeds. Prolonged cooking of aged seeds also reduced the levels of minerals [Mg, Fe, and Zn; (45)] and protein quality (122). The level of HTC and degree of cooking also influenced starch digestibility of HTC Bambara groundnut (115). Additionally, hardening of beans not only reduces the eating quality but also increases fuel and water consumption during cooking (14). This becomes a major problem especially for areas where firewood is used for fuel or water resources are limited. Consequently, the HTC phenomenon is not only inconvenient, it increases the cooking cost and also poses challenges for sustainability, which may limit its potential uptake on a larger scale (115).

Possible solutions to address the HTC defect include improving the storage conditions and breeding cultivars that are less prone to HTC (114). The former may require energy and incur additional costs, which may be infeasible for resource-poor areas; while the latter may take several generations of cross breeding. Simple low-cost processing techniques, such as soaking hard seeds in salt solution, has been demonstrated to reduce the cooking time (47, 123, 124). Sodium chloride, sodium bicarbonate, and alkaline rock salt are among the commonly used salts. Salts are believed to reduce cooking time through several actions: increased pectin solubilization by displacing divalent cations bound to pectin; increased protein solubility through modifying the pH of soaking medium; and enhanced water uptake and thermal penetration by improving bean porosity (116). These actions are influenced by the type, concentration, and monovalent-to-divalent ratio of salt (123, 124).

Further research is required to gain a better understanding of the fundamental mechanisms that lead to the development of HTC traits, and to elicit a viable, long-term solution to the problem.

#### Value Chain Constraints

Throughout Africa, Bambara groundnut is regarded by many as an important form of food security to be relied on when food is scarce (6, 66). Its tasty, nutritious properties are also recognized and valued by consumers, creating demand for the fresh seeds (125). Additionally, various cultural and religious beliefs are associated with consumption of the crop (126, 127). Despite the importance attached to the crop, there remain several obstacles to promoting local utilization of Bambara groundnut in the region. Inadequate (94) and inconsistent supply (128) are among the major barriers to wider consumption. Low market availability of the crop could be due to inefficient farming (129), poor weather and soil (127), competition and displacement by cash-crop farming (130), pests and diseases (5), lack of resources including access to high-quality seeds, land and storage, labor, capital and extension services (125), and societal norms and beliefs that restrict cultivation, retail, and consumption of Bambara groundnut (126). Difficulty to process and prepare due to its HTC and hard-to-mill properties are also reported to be one of the major concerns by consumers (10, 94). Besides, large-scale postharvest processing is further limited by lack of capital, processing facilities, and low product quality (6). Other factors such as disliked by consumers and flatus-causing attribute also limit its local adoption and consumption (125). Local demand and price may fluctuate over the year (6), which might in turn discourage cultivation of the crop.

Seed selection criteria, production constraints, and socioeconomic challenges vary across regions. Therefore, local bodies may play a significant role in developing strategies and policies to effectively pinpoint and address the local challenges. Community biodiversity management through provision of training and education, and enhanced seed access, has been proven to successfully improve adoption of Bambara groundnut in a few regions in Mali (130). Tackling other value chain bottlenecks such as infrastructure, processing unit and market access, and raising awareness about the contribution of Bambara groundnut to agri-food systems, population health, and community welfare, may prove crucial in encouraging more widespread local uptake of Bambara groundnut (130). A complete value chain can help to ensure constant supply and reduced wastage, thereby preventing price fluctuation and enhancing utilization.

There is very little evidence of Bambara groundnut trading outside Africa (5), suggesting that it is relatively unknown by the rest of the world. Promoting the use of Bambara groundnut at global levels through effective promotion strategies may drive demand for the crop, which in turn could encourage cultivation and intervention policies by local and national bodies. In order to increase its popularity worldwide, it would be sensible to first understand consumers' demand and preferences. Several studies indicated sensory attributes being the major determinant of consumer acceptance to novel Bambara groundnut products (41, 67, 94). Certain pretreatments, e.g., dehulling, roasting, germination, and fermentation have been shown to result in higher sensory scores (53, 67). Besides, product formulation is imperative in improving consumer acceptability. Correct formulations and substitution levels could sometimes yield products that are more desirable than conventional products (67, 81). Lack of familiarity with the ingredient could deter consumers, but this aspect could be remedied by incorporating traditional ingredients into product formulation (88, 94). Additionally, pricing of the products should be taken into consideration to ensure they are affordable for most people. Lastly, marketing strategy of these products should be specialized for targeted consumer groups. Innovation, nutritional quality, and agroecological and health-promoting features are among the factors motivating consumers to consume Bambara groundnut products (41, 131).

#### Food Safety and Allergenicity

It is essential that any undesirable, and potentially toxic, attributes of Bambara groundnut are addressed to improve utilization. The presence of *Aspergillus flavus* and its aflatoxin has been detected in Bambara groundnut (132). The study reported that uncontrolled fermentation resulted in the worst proliferation of the fungus, and that although roasting eliminated aflatoxin before storage, it could not suppress fungal growth during storage. This raised concerns about implications of quality control during post-harvest processing, food production and storage, especially in humid tropical areas and regions with limited resources. There is also a need for quality assurance by accredited food testing facilities to ensure compliance with food safety regulations before the product reaches consumers.

Another area of concern is the possible presence of allergenic proteins in Bambara groundnut (133). There is very little reported research specific to Bambara groundnut, but it is essential to determine the magnitude of the problem among the various cultivars and landraces if it is to be adopted and promoted widely as an alternative food or ingredient.

### The Roles of Bambara Groundnut in Closing Nutrient Gap

The nutrition transition in Africa, characterized by a move away from the production and consumption of traditional staple foods, rich in starch and dietary fiber, to more palatable staples and cheap processed food, is one of the drivers of the decline in consumption of Bambara groundnut. Other typical impacts of the nutrition transition include a decrease in plant protein sources, such as legumes, and increased availability and consumption of energy-dense snack foods, carbonated sweetened beverages, added sugar, fats, and oils in food preparation (134). Such changes in dietary pattern are propelled by economic and social development, urbanization, and acculturation, and they affect people of all socioeconomic status. For the rich and those with increased disposable income, the shift to highly palatable refined carbohydrates and animal sourced protein fulfills an aspirational goal. For others, the choice of food is dictated by circumstances. Trade liberalization and increased availability and affordability of ultra-processed food, table sugar, and cooking oil have elevated energy-dense nutrient-free food to be the “food of necessity.” These foods are affordable, palatable, and easy to prepare. Increases in fuel and electricity prices have hampered food preparation and forced households to resort to less nutritious processed food that requires little preparation.

Nutrition transition is associated with increases in non-communicable diseases (NCDs) in developing countries (135). As a result, many of the developing countries, including the poorest, face the multiple burden of malnutrition. Hunger and under-nutrition, of especially energy and several micronutrient deficiencies, have not been successfully addressed in Africa while the epidemiologic transition is seen in the increased prevalence of obesity and other NCDs in many African countries (136, 137). The nutrition transition in developed countries presents well-identified features that help to predict consumption changes in low- and middle-income countries (LMICs) as they go through socioeconomic changes. Taking lessons from intensive monoculture and heavy reliance on major crops in developed economies, as well as their impacts on agricultural sustainability and nutritional status, future policy development in LMICs should place emphasis on diversification of national food supplies, and the access to and affordability of diverse and quality diets.

Countries at different stages of structural transformation should employ different strategies to enhance the contribution of agriculture to diet quality and nutrition (138). Pingali et al. (138) suggested that low-productive agricultural systems should focus on yield enhancement, while maintaining production diversity and ensuring equitable conditions for working women. Diversification of food supplies has been shown to be negatively associated with indicators of the prevalence of undernutrition (139). Climate and soil fertility are also critical to semi-subsistence agricultural production and human welfare in LMICs. As crops are grown for both household consumption and for income, there are multiple connections between these two factors and poverty and the health of the families that work the land. Bambara groundnut, being a climate-resilient and nitrogen-fixing legume, is an ideal candidate crop for diversifying local food production system while improving the nutritional status of the community.

The elevation of Bambara groundnut, from under-utilized to more mainstream crop, would theoretically be less challenging if industry led. By promoting its use at a commercial level, smallholder farmers, while producing enough to feed the family, can sell the surplus to industrial producers for monetary returns that can be then used to improve the quality of life and nutritional status of the family. To increase utilization at the household level, it is important to address the HTC phenomenon. As evidenced by the health benefits detailed above, fortification or substitution of commercial products with Bambara groundnut can enhance the nutrient density of the products. There are numerous potential opportunities that deserve more research before they can be fully exploited. From a commercial point of view, demand for these products has to be created. Changing consumers' perceptions requires creative and effective marketing strategies, as is the case of quinoa (140). Sustainability and equity aspects should be considered and integrated into the entire value chain.

## Conclusion

Despite being a minor crop, Bambara groundnut has the potential to play a role in combating food insecurity and malnutrition at household, national, and global levels. It has high adaptability in various growing conditions and can produce reasonable yield under environmental stresses. Not only does Bambara groundnut fix nitrogen to improve soil health, it also increases crop yield when incorporated into intercropping system. These positive attributes address the supply chain gap by ensuring supply stability and sustainability in the face of climate variability and resource depletion. The genetic diversity across Bambara groundnut landraces allows crop improvement program to select for desirable agronomic, nutritional, and processing traits that are advantageous to improved food and nutritional security.

Knowledge of the detailed nutritional quality and physicochemical attributes of Bambara groundnut will enable the wider use and application of the crop in numerous food products, for instance as alternative flour, modified starch, or protein isolate. The nutritional values, sensory attributes, and functional characteristics of food products can be modified through appropriate processing techniques.

The hard-to-cook phenomenon, value chain bottlenecks, and food safety remain the major constraints to increasing the widespread utilization of Bambara groundnut. These obstacles could be addressed by different players across the value chain through increased collaborative and interdisciplinary approaches in order to realize the full potential of Bambara groundnut's contribution toward food and nutrition security.

## Author Contributions

XT and FM: conceptualization. XT, EG, HC, MM, TM, and SuA-A: methodology and original draft preparation. FM and SM: funding from the University of Nottingham Future Food Beacon of Excellence. All authors: review and editing.

## Conflict of Interest

The authors declare that the research was conducted in the absence of any commercial or financial relationships that could be construed as a potential conflict of interest.
